# Respiratory system compliance during anesthesia induction and postoperative mechanical ventilation needs: An observational study

**DOI:** 10.1002/hsr2.2315

**Published:** 2024-08-12

**Authors:** Yukiko Yamazaki, Yuka Matsuki, Koji Hosokawa, Katsuya Tanaka, Yuko Kawamura, Aiko Tanaka, Kenji Shigemi

**Affiliations:** ^1^ Department of Intensive Care University of Fukui Hospital Fukui Japan; ^2^ Department of Anesthesiology and Reanimatology, Faculty of Medical Sciences University of Fukui Fukui Japan; ^3^ Department of Anesthesia Fukui Prefectural Hospital Fukui Japan; ^4^ Department of Anesthesiology and Intensive Care Medicine Osaka University Graduate School of Medicine Suita Japan

**Keywords:** mechanical ventilation, mortality, respiratory system compliance, surgery

## Abstract

**Background and Aims:**

Respiratory system compliance (Crs) is a simple indicator of lung flexibility. However, it remains unclear whether a low Crs during anesthesia induction (iCrs) is associated with an increased risk of postoperative mechanical ventilation.

**Methods:**

This retrospective observational study was conducted using a local database. All mechanically ventilated postoperative ICU patients were included in this study. The duration of postoperative mechanical ventilation, length of hospital stay, and in‐hospital mortality were compared between the low iCrs group (<25% of distribution) and the normal iCrs group.

**Results:**

A total of 315 patients were classified into the low iCrs (<39 mL/cmH_2_O) group (*n* = 78) or the normal iCrs group (*n* = 237). Low iCrs was associated with a higher chance of mechanical ventilation in 28 days (log‐rank test, *p* < 0.001). The duration of hospital stay was similar. Multivariate analysis showed that in‐hospital mortality was higher in the low iCrs group than in the normal iCrs group (adjusted odds ratio, 6.04 [1.13, 32.26]; *p* = 0.04).

**Conclusion:**

Low iCrs was associated with an increased risk of requiring postoperative mechanical ventilation. An additional result of poor survival related to low iCrs may require further study.

## 1 INTRODUCTION

Mechanical ventilation is essential during the perioperative period of major surgeries under general anesthesia. Most patients are relieved from mechanical ventilation immediately after surgery; however, some require postoperative prolonged intubation or reintubation. For the prevention of postoperative pulmonary complications (PPC), several risk factors for postsurgical mechanical ventilation have been summarized to date.^1^, ^2^ Among them, forced expiratory volume in 1 s (FEV_1.0_) has been described as one of the most important parameters to predict PPCs, indicating preoperative respiratory frailty in patients.^3^, ^4^ However, pulmonary function tests may not be feasible for certain surgical patients, such as those undergoing emergency procedures.

In determining lung physics, respiratory system compliance (Crs), normally defined as the tidal volume divided by the difference between plateau pressure and positive end‐expiratory pressure (PEEP), suggests the flexibility of the lung for positive pressure ventilation.^5^, ^6^ Managing Crs during surgery by adjusting PEEP or performing lung recruitment has been linked with a decreased incidence of PPCs.^7^, ^8^ However, effectively managing Crs during surgery can be challenging due to factors such as abdominal pressure due to laparotomy, one‐lung ventilation, or leg‐raising positioning. Conversely, the respiratory parameter at the induction of anesthesia, an original measure of an individual patient, was independently associated with the duration of postoperative composite oxygen therapy.^9^ However, the relationship between the postoperative requirement of mechanical ventilation and the Crs during anesthesia induction (iCrs) has not been thoroughly evaluated. Therefore, this study aimed to investigate whether patients with low iCrs have an increased risk of requiring mechanical ventilation postoperatively.

## 2 METHODS

We conducted a secondary analysis of previous retrospective observational study.^9^ Patients were included from January 1, 2019, to December 31, 2020. Ethical approval for this study was obtained from the Research Ethics Committee of Fukui University (#20210023). The requirement for written informed consent from participants was waived and, as an opt‐out policy, the study information was cited on the hospital website, and the participants were allowed to deny their inclusion in the study via direct contact with the researchers. We followed STrengthening the Reporting of Observational studies in Epidemiology (STROBE) guideline.^10^

### 2.1 Patients

All patients who received mechanical ventilation in the ICU 28 days after surgery were included in the database. Patients who did not have any iCrs were excluded. Patients were then categorized into low iCrs (<25% of the distribution) and normal groups. Despite the possibility that it may be a poor breaking point, this cut‐off number was predetermined as a mathematical quadrant. The details regarding anesthesia induction and respiratory management are reported as supplemental materials.

### 2.2 Database and iCrs measurement

Crs was basically dynamic compliance under pressure controlled ventilation that was displayed on Aisys CS 2 using manufacture‐driven calculations. Details of methods were described previous report.^9^

### 2.3 Outcomes

The primary outcome was the need for postoperative mechanical ventilation within 28 days. Secondary outcomes were re‐intubation rate, duration of composite oxygen support, length of ICU or hospital stay, and in‐hospital mortality.

### 2.4 Statistical analysis

As for sample size, it was estimated that 387 persons were required when an *α* of 0.05 and a detection rate of 80% was assumed for a two increase in the seven of standard deviation. Continuous data was presented as medians and interquartile ranges, and categorical data is presented as numbers and percentages. For nonparametric univariate analysis, the patients' demographics, duration of mechanical ventilation or other clinical values of the two groups were compared using the Wilcoxon test or Fisher's exact test. In addition, the duration of mechanical ventilation in the two groups was compared and tested using Kaplan–Meier curves and log‐rank test, respectively. For multivariate analysis, the parameters that were significantly different between the two iCrs groups (low: normal) were examined using the hazard ratio for continuous values and a logistic regression model for dichotomous data to calculate the odds ratio. For these analyses, age (years), sex (male and female), American Society of Anesthesiologists physical status (1, 1E, 2, 2E, 3, 3E, >4, and >4E), duration of surgery (min), and surgical category (10 categories) were used as explanatory factors. Sensitivity analysis was conducted according to the textbook by Lash.^11^ Assuming that the effect of unknown confounders varied from an odds ratio of 0.1−10, and that the frequency of unknown confounders varied from equal to twice as much, adjusted odds ratios were calculated.

The combined database was cleaned or processed using Microsoft Excel version 2301 on Microsoft 365 (Microsoft, Redmond, WA). Statistical analyses were performed using JMP 17 Pro software (SAS). A two‐sided *p* Value of 0.05 was considered significant.

## 3 RESULTS

**Figure 1:**
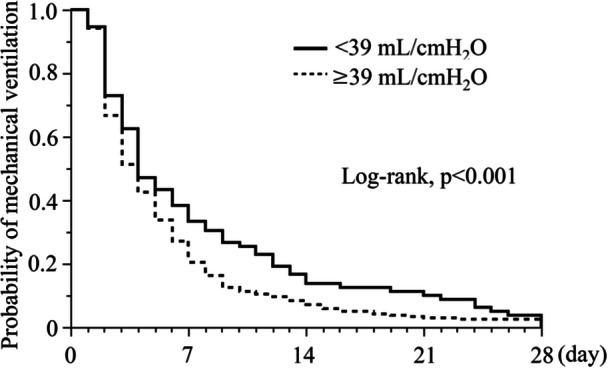
The probability of mechanical ventilation between two compliance groups. The incidence of mechanical ventilation is compared between the two groups of patients with respiratory system compliance during anesthesia induction for surgery (iCrs). The low iCrs is defined as <39 mL/cmH_2_O. Kaplan–Meier curves demonstrate a significant difference between the groups (*p* < 0.001, log‐rank test).

Among the 5607 surgical patients during the study period, 315 (5.6%) were included (Supporting Information S1: Figure 1). The patients were divided into two groups according to distribution of iCrs (iCrs < 39 mL/cmH_2_O, *n* = 78; iCrs ≥ 39 mL/cmH_2_O, *n* = 237) (Supporting Information S1: Figure 2 and Table 1). Patient characteristics were remarkably different between the groups (Table 1). Interestingly, the rate of emergent surgery in the low iCrs group was double than that in the normal group (53% vs. 25%, *p* < 0.001; Table 1).

**Table 1:** Patient characteristics.

		All case (*n* = 315)	Patient group		
			iCrs < 39 mL/cmH_2_O (*n* = 78)	iCrs ≥ 39 mL/cmH_2_O (*n* = 237)	*p* Value
Age (year)		69 [58, 77]	74 [61, 83]	68 [57, 74]	0.002
Sex (Male: Female)		201 (64%): 114 (36%)	29 (37%): 49 (63%)	172 (73%): 65 (27%)	<0.001
Body mass index (kg∙m^−2^)		22.4 [20.0, 25.3]	22.8 [19.8, 25.7]	22.3 [20.1, 25.1]	0.94
ASA physical status					<0.001
	1, 1E	2 (1%), 2 (1%)	0 (0%), 0 (0%)	2 (1%), 2 (1%)	
	2, 2E	91 (29%), 11 (3%)	15 (19%), 4 (5%)	76 (32%), 7 (3%)	
	3, 3E	122 (39%), 85 (27%)	22 (28%), 36 (46%)	100 (42%), 49 (21%)	
	≥4, ≥4E	0 (0%), 2 (1%)	0 (0%), 1 (1%)	0 (0%), 1 (1%)	
Emergency hospital admission		119 (38%)	42 (54%)	77 (32%)	<0.001
Emergency surgery		100 (32%)	41 (53%)	59 (25%)	<0.001
Surgical category					0.009
	Chest, cardiovascular	128 (41%)	30 (38%)	76 (32%)	
	Abdominal	113 (36%)	37 (47%)	98 (41%)	
	Head, neck^a^	56 (18%)	6 (8%)	50 (21%)	
	Extremity, spine	12 (4%)	2 (3%)	10 (4%)	
	Other	6 (2%)	3 (4%)	3 (1%)	
Laparoscopic procedure		21 (7%)	4 (5%)	17 (7%)	0.79
One‐lung ventilation		28 (9%)	8 (10%)	20 (8%)	0.65
Duration					
	Anesthesia	564 [372, 856]	408 [238, 597]	640 [426, 914]	<0.001
	Surgery	420 [241, 708]	296 [132, 443]	488 [319, 801]	<0.001

*Note*: Values are presented as percentages or medians [interquartile ranges].

Abbreviations: ASA, American Society of Anesthesiologists; iCrs, respiratory system compliance at induction of anesthesia.

^a^ Including the larynx.

The reintubation rate in the low iCrs group was twice as high as that in the normal group; however, the difference was not statistically significant (Table 2). The likelihood of mechanical ventilation in the low iCrs group was higher than that in the normal group (log‐rank test, *p* < 0.001; Figure 1), whereas the univariate analysis revealed no statistically significant difference (Table 2). The low iCrs group had a longer ICU stay than the normal group (8^4^, ^12^ vs. 6,^4^, ^9^ *p* = 0.013) and smaller in‐hospital mortality (6 [7.7%] vs. 5 [2.1%], *p* = 0.03). Since the differences in background between the groups were large, we conducted multivariate analysis and sensitivity analysis. Multivariate analysis showed that the difference in ICU stay disappeared (hazard ratio, 0.97 [0.72, 1.32]; *p* = 0.89), and in‐hospital mortality in the low iCrs group was higher than that in the normal group (odds ratio, 6.04 [1.13, 32.26]; *p* = 0.04). Sensitivity analysis revealed that the adjusted Mantel‐Haenszel odds ratio in in‐hospital mortality varied from 2.28 to 5.91, when the effect of unknown confounders varies from an odds ratio of 0.1 to 10, and the frequency of unknown confounders varies from half to twice as much.

**Table 2:** Outcomes.

	All cases (*n* = 315)	Patient group		
		iCrs < 39 mL/cmH_2_O (*n* = 78)	iCrs ≥ 39 mL/cmH_2_O (*n* = 237)	*p* Value
Prolonged ventilation after surgery	297 (94%)	71 (91%)	226 (72%)	0.13
Mechanical ventilation (day)	4 [2, 7]	4 [2, 11]	4 [2, 7]	0.05
Ventilator free day in 28 days (day)	24 [21, 26]	24 [17, 26]	24 [21, 26]	0.05
Reintubation	24 (7.7%)	9 (11.5%)	15 (6.3%)	0.15
Composite oxygen support (day)	8 [5, 14]	10 [5, 17]	8 [5, 13]	0.11
ICU stay (day)	7 [4, 10]	8 [4, 15]	6 [4, 9]	0.013
Hospital stay (day)	31 [24, 47]	33 [24, 45]	30 [24, 47]	0.88
In‐hospital mortality	11 (3.5%)	6 (7.7%)	5 (2.1%)	0.03

*Note*: Values are presented as percentages or medians [interquartile ranges]. Univariate analysis was conducted to show *p* Value.

Abbreviations: iCrs, respiratory system compliance at induction of anesthesia; ICU, intensive care unit.

## 4 DISCUSSION

The current study found that low iCrs was associated with a higher risk of postoperative mechanical ventilation than normal iCrs. Additionally, we demonstrated through multivariate analysis that in‐hospital mortality was higher in the low iCrs group than in the normal group.

Recently, intraoperative respiratory management aimed at reducing PPCs has shown encouraging outcomes.^9^, ^13^ Lung protective ventilation has been introduced not only in severe respiratory diseases but also in surgical patients, and adequate PEEP was associated with a lower incidence of PPCs.^7^, ^8^, ^14^, ^15^ The recruitment maneuver also prevents atelectasis and diminishes PPCs.^12^ Patients who experienced PPC required mechanical ventilation and a prolonged hospital stay, which could result in increased healthcare costs. While PPCs encompass a variety of conditions, such as pneumonia, atelectasis, pulmonary embolism, and acute respiratory distress syndrome, the duration of mechanical ventilation could be a surrogate parameter suggesting a clinically severe conditions such as PPCs.^16^ In the current study, we hypothesized that low initial Crs before surgery may be associated with a higher chance of mechanical ventilation.

Furthermore, we found that low iCrs is associated with poor survival. Given that driving pressure is a strong predictor of mortality in acute respiratory distress syndrome,^17^ it is reasonable to speculate that the original Crs of surgical patients influence outcomes. From the data of a wide range of surgical patients, we previously showed that a higher driving pressure is associated with a longer postoperative duration of oxygen support and higher mortality.^9^ The current study limited patients to those who required mechanical ventilation 28 days postoperatively and demonstrated the contribution of iCrs to postsurgical survival. One may argue that the difference in characteristics between the groups was significant; 50% of low Crs group patients included the emergency surgical patients. Therefore, adjustment of comparisons by preoperative and intraoperative confounders using multivariate analysis was conducted. We demonstrated a significant association between low iCrs and in‐hospital mortality. These findings warrant further investigation with other methodologies.

This study had several limitations. First, Crs values were not obtained by seeking adequate respiratory settings using a strict protocol due to the retrospective observational design of the research. Additionally, they may have been affected by atelectasis due to various factors such as mask ventilation, apnea time, and dose of muscle relaxants since our data did not include whether the recruitment maneuver was applied during this period or not. Second, the single‐minute values of the Crs contained outliers. The mathematical removal of outliers reduces the reliability of a value. Third, regarding outcome comparisons, a single‐center, and small study population did not gain generalized notice. Additionally, we did not calculate the sample size that would have significant statistical power to detect differences in outcomes. Fourth, our study contained mixed populations and we did not obtain usual preoperative assessments for predicting PPCs; therefore, the results could not be compared with the general incidence of PPCs in other populations. Fifth, we could not deny that the duration of mechanical ventilation would be affected by other implicit preoperative patient's pulmonary factors, in addition to iCrs, since we lacked the results of pulmonary function tests and were not fully informed about lung physiology except for respiratory parameters at the induction of anesthesia. Sixth, despite the statistical relationship between iCrs and mortality, other significant causal factors probably contributed to mortality. If we could examine the diverse perioperative causative factors, the impact of low iCrs on mortality may become clearer. Finally, Crs after induction of anesthesia is not something that can be modified by respiratory settings; however, this physiological knowledge warns clinicians to perform strict observation and treatment.

In conclusions, low iCrs was associated with an increased risk of mechanical ventilation events after surgery. A possible link between low iCrs and high mortality rates has also been suggested. The importance of iCrs should be considered in emergency surgical patients who do not undergo preoperative examinations of lung function.

## AUTHOR CONTRIBUTIONS

**Yukiko Yamazaki**: Formal analysis, visualization, and roles/writing—original draft. **Yuka Matsuki**: Formal analysis, visualization, and writing—reviewing and editing. **Koji Hosokawa**: Conceptualization, methodology, formal analysis, visualization, validation, and writing—reviewing and editing. **Yuko Kawamura**: Visualization, writing—reviewing and editing. **Katsuya Tanaka**: Data curation, validation, and writing—reviewing and editing. **Aiko Tanaka**: Methodology, and writing—reviewing and editing. **Kenji Shigemi**: Supervision, and writing—reviewing and editing. All authors have read and approved the final version of the manuscript. Koji Hosokawa had full access to all of the data in this study and takes complete responsibility for the integrity of the data and the accuracy of the data analysis.

## CONFLICT OF INTEREST STATEMENT

The authors declare no conflict of interest.

## ETHICS STATEMENT

Research involving human data was performed in accordance with the Declaration of Helsinki. Ethical approval for this study was obtained from the Research Ethics Committee of Fukui University (approval no. #20210023). The requirement for written informed consent from participants was waived by the Research Ethics Committee of Fukui University because of the retrospective nature of the study. However, as an opt‐out policy, the study information was cited on the hospital website, and the participants were allowed to deny their inclusion in the study via direct contact with the researchers.

## TRANSPARENCY STATEMENT

Koji Hosokawa affirms that this manuscript is an honest, accurate, and transparent account of the study being reported; that no important aspects of the study have been omitted; and that any discrepancies from the study as planned (and, if relevant, registered) have been explained.

## Supporting information

Supporting information.

## Data Availability

The data sets generated and/or analyzed during the current study are not publicly available to avoid unintended use, but are available from the corresponding author on reasonable request.
