# Supplementary material for: Respiratory system compliance during anesthesia induction and postoperative mechanical ventilation needs: An observational study
Source: Health Sci Rep. 2024 Aug 12;7(8):e2315. doi: 10.1002/hsr2.2315 (PMC11319399; doi:10.1002/hsr2.2315)
Supplement: Supplementary file 1 — Supporting information. [file HSR2-7-e2315-s001.docx]

Supplementary file

Respiratory system compliance during anesthesia induction and postoperative mechanical ventilation needs: An observational study

Yukiko Yamazaki, Yuka Matsuki, Koji Hosokawa, Katsuya Tanaka, Yuko Kawamura, Aiko Tanaka Kenji Shigemi

Supplementary document of methods. Additional information of respiratory management

1. Anesthesia induction and respiratory settings

As a routine protocol, general anesthesia was induced using propofol (target control infusion, 2.0‒3.5 microg/ml) or midazolam (0.4‒1.2 mg/kg) and remifentanil (0.15‒0.3 microg/kg/min) with rocuronium (0.6‒1 mg/kg). Mask ventilation was continued until train-of-four counts reached 0 or 1. A minimal apneic interval was followed by tracheal intubation.

The respiratory setting was determined by admitted anesthetists. The volume-guaranteed pressure control ventilation (PCV-VG) were used as the routine respiratory mode on Aisys CS 2. Tidal volume of respiration was set 7‒10 × body weight (mL) and PEEP were set 5 cmH_2_O. We had not established a role that the recruitment maneuver was applied immediately after tracheal intubation.

Crs was displayed on Aisys CS 2 using manufacture-driven calculations. As the manufacture answered, the calculation formula is followed; in case of pressure control ventilation, the value of dynamic Crs used; Crs = tidal volume/(maximum pressure‒PEEP)]. Other respirator mode and parameters were also obtained and stored in GAIA anesthetic chart system. iCrs (Crs just after tracheal intubation while the induction of anesthesia was performed) was the mean of nine consecutive minute values after tracheal intubation.

Supplementary Table 1. Initial respiratory parameters

|  | All cases (n=315) | Patient group | |  |
| --- | --- | --- | --- | --- |
|  |  | iCrs <39 mL/cmH_2_O (n=78) | iCrs ≥39 mL/cmH_2_O (n=237) | p value |
| From start of anesthesia to intubation (min) | 9 [8-12] | 9 [8-12] | 9 [8-12] | 0.95 |
| Mode of respiration; PCV-VG:PCV:Other (%) | 92%:2%:6% | 88%:3%:9% | 93%:2%:5% | 0.43 |
| Tidal volume (mL) | 399 [350-452] | 351 [303-399] | 409 [363-464] | <0.001 |
| Tidal volume/body weight (mL/kg) | 6.6 [6.1-7.4] | 6.5 [6.0-7.3] | 6.7 [6.1-7.4] | 0.05 |
| Tidal volume/predicted body weight (mL/kg) | 9.2 [7.9-10.8] | 10.2 [8.3-12.1] | 9.1 [7.9-10.3] | 0.002 |
| Peak airway pressure (cmH₂O) | 14.8 [13.1-16.5] | 16.9 [14.9-18.8] | 14 [12.9-15.7] | <0.001 |
| PEEP (cmH₂O) | 4.7 [4.0-5.1] | 4.6 [3.8-5.0] | 4.8 [4.1-5.1] | 0.30 |
| Driving pressure (cmH₂O) | 8.6 [6.8-8.6] | 11.2 [9.3-13.2] | 7.7 [6.2-9.2] | <0.001 |
| Crs (mL/cmH₂O) | 48 [39-61] | 32 [29-36] | 53 [45-64] | <0.001 |

iCrs, respiratory system compliance at induction of anesthesia; PCV-VG, pressure control ventilation, volume guarantee; PEEP, positive end-expiratory pressure.

2. Postoperative respiratory managements

The criteria that patients were not extubated from trachea in the operation theater were; high risk of postoperative bleeding (underwent cardio-pulmonary bypass, carotid artery surgery), larynx or mandibular surgery with plastic surgery, and delayed consciousness after cessation of sedatives. After certain periods of postoperative observation, tracheal extubation was decided with general clinical condition and no abnormal signs with spontaneous awaking and breathing trials.

Supplementary Figure 1. Study patient flow


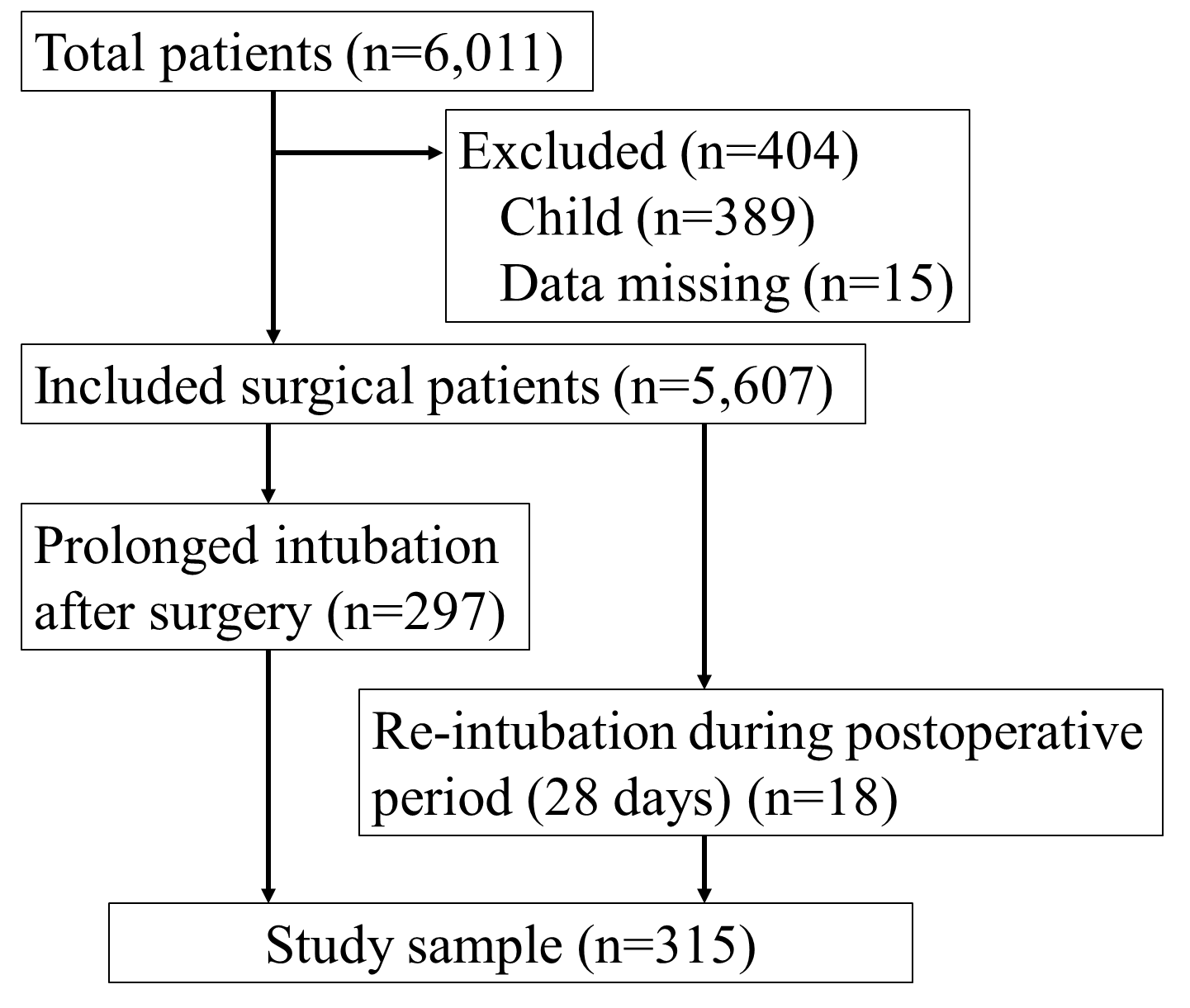


Supplementary Figure 2. Distribution of respiratory system compliance during anesthesia induction for surgery (iCrs)





iCrs, respiratory system compliance during anesthesia induction for surgery.
